# Factors associated with high influenza vaccination among healthcare workers in Tennessee acute-care hospitals, 2014–2022

**DOI:** 10.1017/ash.2023.323

**Published:** 2023-09-29

**Authors:** Ashley Gambrell, Raquel Villegas, Christopher Wilson, Simone Godwin

## Abstract

**Background:** Healthcare workers (HCWs) are at increased risk of influenza exposure and represent a potential transmission source. The Department of Health and Human Services (HHS) set a goal for 2020 to have 90% of all HCWs in acute-care hospitals (ACHs) vaccinated. Vaccination against influenza decreases symptomatic illness and absenteeism and protects HCWs and their contacts. We assessed characteristics of facility intervention programs based on their success in meeting this benchmark. **Methods:** Data from the NHSN were utilized, including answers to the Annual Flu Survey for 2014–2022 and the rate of vaccine compliance by facility. Flu surveys detail facility-specific programs implemented for each influenza season, from October to March. We used SAS version 9.4 software for univariate analyses to determine factors significantly associated with meeting the HHS benchmark target of ≥90% vaccination among all HCWs, split into categories for employees, students or volunteers, and licensed independent practitioners. Facilities were excluded if they were not ACHs or Critical Access Hospitals (CAH), did not complete the Annual Flu Survey for at least 1 year, or required vaccination as a condition of employment. **Results:** From 2014 to 2022, 745 surveys were completed. Overall, 48.58% of respondents succeeded in meeting the HHS benchmark. Also, 306 surveys completed noted that their facility did not require influenza vaccination. Among those, only 19.93% respondents succeeded. Moreover, 80.33% of successful respondents for all HCWs required personal protective equipment (PPE) upon vaccination refusal compared to 34.29% of unsuccessful respondents (*P* < .0001). Furthermore, 98.36% successful respondents required documentation of offsite vaccination, compared to 89.39% of unsuccessful respondents (*P* = .027). For employees, 64.56% of successful respondents tracked vaccination rates in some or all units compared to 45.81% of unsuccessful respondents (*P* = .004). Also, 63.29% successful respondents had visible vaccination of leadership, compared to 43.61% of unsuccessful respondents (*P* = .003). Furthermore, 86.08% of successful respondents had mobile vaccination carts, compared to 73.57% unsuccessful respondents (*P* = .023). For the student- or volunteer-specific benchmark, 24.59% of successful respondents provided vaccination incentives compared to 14.63% of unsuccessful respondents (*P* = .035). **Conclusions:** Facilities with ≥90% vaccination among HCWs were more likely to require PPE after vaccination refusal and documentation for offsite vaccination. Other strategies for vaccination were differentially associated by employee type for Tennessee facilities. For future outreach, a multipronged approach is more likely to be successful in addressing vaccine uptake among employees with lagging rates. Strategies for influenza vaccine uptake could also improve other occupational vaccinations. More research is needed on the barriers to vaccination among HCWs specifically.

**Disclosures:** None

